# Supplementary material for: Anti-Biofilm Activity of a Hyaluronan-like Exopolysaccharide from the Marine Vibrio MO245 against Pathogenic Bacteria
Source: Mar Drugs. 2022 Nov 21;20(11):728. doi: 10.3390/md20110728 (PMC9696739; doi:10.3390/md20110728)
Supplement: Supplementary file 1 [file marinedrugs-20-00728-s001.zip › marinedrugs-2014781 - supplementary.pdf]

marine drugs

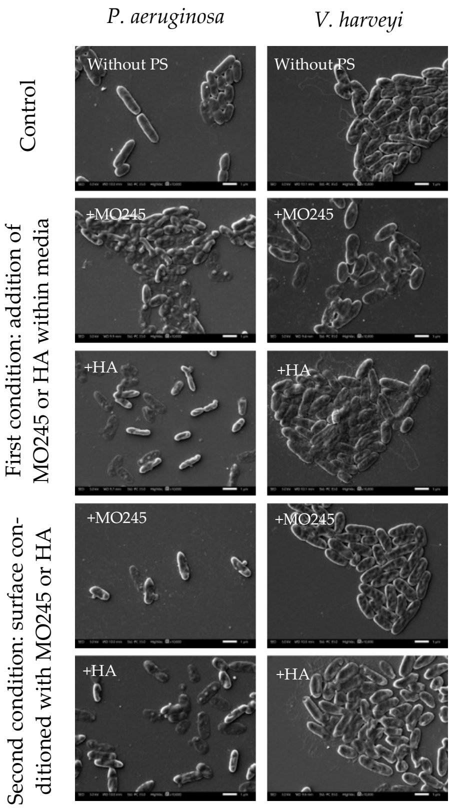

Figure S1. SEM images of the morphology of *P. aeruginosa* and *V. harveyi* without or with addition of MO245 or HA to the medium during adhesion or with surface conditioning by MO245 or HA before bacterial adhesion. MO245 or HA were used at 125 µg/mL and bacteria adhered 2 h at 20 °C. Bacteria were observed by SEM after fixation with glutaraldehyde and dehydration with ethanol.
